# First record of the genus *Cephalispa* Malloch (Diptera, Muscidae) in Korea, with an identification key to East Asian species

**DOI:** 10.3897/BDJ.14.e195650

**Published:** 2026-08-11

**Authors:** Dongmin KIM, Sang Jae Suh

**Affiliations:** 1 Advanced Bio-resource Research Center, Kyungpook National University, Daegu, Republic of Korea Advanced Bio-resource Research Center, Kyungpook National University Daegu Republic of Korea; 2 Department of Plant Medicine, Kyungpook National University, Daegu, Republic of Korea Department of Plant Medicine, Kyungpook National University Daegu Republic of Korea

**Keywords:** *Cephalispa acerca*, identification key, Korea, Muscidae, predatory behaviour

## Abstract

**Background:**

The genus *Cephalispa* Malloch, 1935 (Diptera, Muscidae, Coenosiinae) includes 32 species worldwide and is mainly distributed in the Oriental and Afrotropical Regions, with few records from the Palaearctic Region. The genus remains poorly known and has not previously been recorded from Korea.

**New information:**

*Cephalispa acerca* (Xue, Wang & Ni., 1990) is recorded from Korea for the first time, representing the first national record of the genus and the easternmost record of the species. The male is re-described and the female is described for the first time. An identification key to the males of East Asian species is provided. Predatory behaviour was repeatedly observed under natural conditions, with individuals capturing small insects using an ambush strategy on herbaceous leaves. This represents the first ecological information for the genus. These findings improve the understanding of the taxonomy, distribution and ecology of *Cephalispa* in East Asia.

## Introduction

The genus *Cephalispa* Malloch, 1935 (Diptera, Muscidae, Coenosiinae) was originally established as a subgenus of *Lispocephala* Pokorny, 1893, with *C. scutellata* (Malloch, 1935) designated as the type species. It was subsequently elevated to generic rank by Crosskey (1962), based on its distinct morphological characteristics within the subfamily Coenosiinae.

The genus is widely distributed in tropical and subtropical regions of the Old World, with the majority of species recorded from the Oriental Region. Currently, 32 valid species are recognised worldwide ( Xue et al. 1989, Cui et al. 1995, Couri et al. 2006, Wei and Yang 2007, Yoshizawa and Tachi 2016 ). Most species occur in the Oriental Region, particularly in southern China, Southeast Asia and adjacent areas, whereas comparatively few species are known from the Palaearctic Region. This distribution pattern suggests that the genus is primarily associated with warm and humid environments.

Although *Cephalispa* has not previously been recorded from Korea, its occurrence has been anticipated given the proximity of the Korean Peninsula to known localities in north-eastern China and Japan. The absence of earlier records is likely attributable to insufficient sampling rather than true absence.

In the present study, we report *Cephalispa* from Korea for the first time, based on specimens of *C. acerca* (Xue, Wang & Ni, 1990). We provide a detailed re-description of the male and describe the female for the first time. An identification key to the males of 20 East Asian species is also provided. This study contributes to improving the taxonomic knowledge of Muscidae in Korea and enhances our understanding of the diversity and biogeography of *Cephalispa* in East Asia.

## Materials and methods

Specimens were examined using a stereomicroscope (Olympus SZX16; Olympus Corporation, Tokyo, Japan) and a compound microscope (Olympus BX50; Olympus Corporation, Tokyo, Japan). External morphological features and genital structures were photographed using a Michrome 16 CMOS camera (Tucsen Photonics Co., Ltd., Fujian, China). Images from multiple focal planes were stacked using Helicon Focus (HeliconSoft Ltd., Kharkiv, Ukraine) and subsequently processed using Adobe Photoshop CS5 Extended (version 12.1 x64; Adobe Systems Inc., San Jose, CA, USA).

Ecological photographs were taken using a Galaxy S23 Ultra (Samsung Electronics, Suwon, South Korea) with the Expert RAW mobile application (Samsung Electronics, Suwon, South Korea) and post-processing was performed using Adobe Lightroom Mobile (version 11.2; Adobe Systems Inc., San Jose, CA, USA).

For genital examination, the abdomens of males and females were dissected after maceration in a 10% potassium hydroxide (KOH) solution at room temperature for about 12 hours, rinsed in distilled water and preserved in glycerine.

All examined specimens are deposited in the collection of the Department of Plant Medicine, Kyungpook National University (KNU), Daegu, Republic of Korea.

## Taxon treatments

### Cephalispa

Malloch, 1935

D05008B6-4D9D-52D0-AA9C-F0BB219D7B40

Cephalispa Malloch 1935: 658 (originally proposed as a subgenus of *Lispocephala* Pokorny).
Status: Elevated to genus rank by Crosskey (1962).
Lispocephala (Cephalispa) scutellata Malloch, 1935

#### Diagnosis

*Head*. Frons at vertex narrow, not exceeding one-fourth of head width, distinctly widened anteriorly; ocellar triangle usually not extending beyond mid-length of frons; ocellar setae reduced to short setulae. Two reclinate orbital setae, two inclinate frontal setae. Antenna with postpedicel approximately twice as long as pedicel; arista plumose to apex. Prestomal teeth well developed.

*Thorax*. Two presutural dorsocentral setae present, the anterior one usually very small or occasionally absent; katepisternal setae 1:1:1, arranged in a triangle, with the lower seta typically equidistant from the two upper setae. Scutellum with well-developed apical and basal setae, the apical seta reduced in *C. curta* Couri.

*Legs*. Fore tibia usually lacking a submedian posterior or posteroventral seta; mid-femur with one pre-apical posterior seta; mid-tibia typically bearing two posterodorsal setae; hind tibia with one anteroventral seta and strong median anterodorsal and posterodorsal setae (Cui et al. 1995, Couri et al. 2006).

#### Distribution

Palaearctic, Oriental, Australasian and Afrotropical Regions.

#### Notes

The genus *Cephalispa* is morphologically similar to *Lispocephala* Pokorny, 1893 and *Pygophora* Schiner, 1868 in general external appearance, which has historically led to taxonomic confusion. However, it can be distinguished from *Lispocephala* by the presence of two posterodorsal setae on the mid-tibia, a relatively narrow frons and a smaller frontal triangle (Cui et al. 1995, Couri et al. 2006). From *Pygophora*, it differs in having reduced, hair-like ocellar setulae, the absence of a submedian posteroventral seta on the fore tibia and a distinct arrangement of katepisternal setae (Crosskey 1962). These characters provide reliable diagnostic criteria for delimiting *Cephalispa* within Coenosiinae.

Korean vernacular name: 노랑범집파리(No-rang-beom-jip-pa-ri)속 (신칭).

### Cephalispa acerca

(Xue, Wang & Ni, 1990)

9B7D212B-BB1E-5F2D-B85A-3BB022B81159

Caricea acerca Xue, Wang & Ni, 1990: 547. Type locality: China. Shaanxi, Mt. Taibai.

#### Materials

- **Type status:** Other material. **Occurrence:** recordedBy: SJ Suh; sex: 1 female; lifeStage: adult; occurrenceID: B4E25B46-2187-508F-A502-DCA8E4769B5D; **Location:** locationID: Mt. Sageum; country: Republic of Korea; countryCode: KR; stateProvince: *Gangwon-do*; county: Samcheok-si; municipality: Icheon-ri, Wondeok-eup; locality: Mt. Sageum; decimalLatitude: 37.206274; decimalLongitude: 129.252542; geodeticDatum: WGS84; **Event:** year: 2018; month: 8; day: 23
- **Type status:** Other material. **Occurrence:** recordedBy: SJ Suh; sex: 3 males, 12 females; lifeStage: adult; occurrenceID: B4E25B46-2187-508F-A502-DCA8E4769B5D; **Location:** locationID: Mt. Geomang; country: Republic of Korea; countryCode: KR; stateProvince: *Gyeongsangnam-do*; county: Hamyang-gun; municipality: Daenam-ri, Seosang-myeon; locality: Mt. Geomang; decimalLatitude: 35.688496; decimalLongitude: 127.721025; geodeticDatum: WGS84; **Event:** year: 2022; month: 7; day: 23
- **Type status:** Other material. **Occurrence:** recordedBy: SJ Suh; sex: 7 males, 9 females; lifeStage: adult; occurrenceID: B4E25B46-2187-508F-A502-DCA8E4769B5D; **Location:** locationID: Mt. Muhak; country: Republic of Korea; countryCode: KR; stateProvince: *Gyeongsangnam-do*; county: Changwon-si; municipality: Wongye-ri, Naeseo-eup; locality: Mt. Muhak; decimalLatitude: 35.226601; decimalLongitude: 128.517636; geodeticDatum: WGS84; **Event:** year: 2024; month: 6; day: 1
- **Type status:** Other material. **Occurrence:** recordedBy: SJ Suh; sex: 1 male, 1 female; lifeStage: adult; occurrenceID: B4E25B46-2187-508F-A502-DCA8E4769B5D; **Location:** locationID: Mt. Gujeol; country: Republic of Korea; countryCode: KR; stateProvince: *Gyeongsangnam-do*; county: Goseong-gun; municipality: Naegok-ri, Donghae-myeon; locality: Mt. Gujeol; decimalLatitude: 35.023380; decimalLongitude: 128.420293; geodeticDatum: WGS84; **Event:** year: 2024; month: 8; day: 9
- **Type status:** Other material. **Occurrence:** recordedBy: Dongmin Kim; sex: 1 female; lifeStage: adult; occurrenceID: B4E25B46-2187-508F-A502-DCA8E4769B5D; **Location:** locationID: Mt.Cheorambagi; country: Republic of Korea; countryCode: KR; stateProvince: *Jeollanam-do*; county: Gwangyang-si; municipality: Chusan-ri, Ongnyong-myeon; locality: Mt.Cheorambagi; decimalLatitude: 35.031919; decimalLongitude: 127.605096; geodeticDatum: WGS84; **Event:** year: 2022; month: 9; day: 11

#### Description

**Figure 1.**
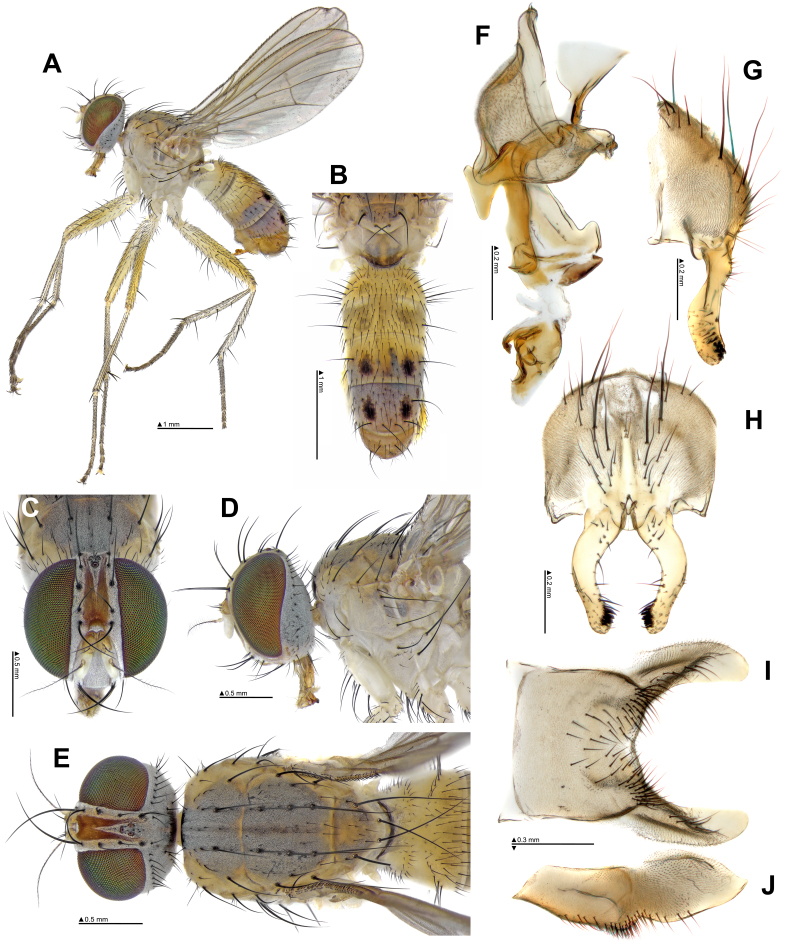
*Cephalispa acerca* (Xue, Wang & Ni, 1990), male. **A** habitus; **B** abdomen, dorsal view; **C** head, frontal view; **D, E** head and thorax, lateral view (D) and dorsal view (E); **F** aedeagus, lateral view; **G, H**, epandrium, surstylus and cercus; lateral view (G) and dorsal view (H); **I, J** sternite 5: ventral view (I) and lateral view (J).

**Male (Fig. 1)**. Body length 4.5–6.2 mm; wing length 4.0–5.3 mm.

Head (Fig. 1C and D). Ground colour yellow to beige. Frons, parafrontalia, face, gena and vertex covered with creamy pruinosity; occiput with greyish pruinosity. Eyes bare, dichoptic; eye obovate in lateral view, posterior margin concave (Fig. 1D). Frons narrowing towards vertex; width at narrowest point approximately 0.25 of head width, at widest point 0.33–0.35 (Fig. 1C). Frontal vitta yellowish-brown; ocellar triangle extending to upper half of frons (Fig. 1C). Ocellar setae absent (Fig. 1D). Two orbital setae, reclinate; two frontal setae, inclinate (Fig. 1C and D). Antenna yellowish-white; pedicel about half as long as postpedicel; postpedicel width about 0.4 of its length. Arista shortly plumose, longest setulae about three times width of aristal base. Parafacial bare (Fig. 1C and D). Epistomal ridge not projecting beyond vibrissal angle in lateral view. Genal height about twice width of aristal base. Palpus slender, yellowish-white. Prementum glossy (Fig. 1D).

Thorax (Fig. 1D and E). Ground colour yellow to beige; thoracic dorsum extending from postpronotal lobe to postalar callus and dorsal surface of scutellum, black and covered with yellowish-white pruinosity. Scutum with three indistinct longitudinal brown vittae along rows of acrostichal setulae and dorsocentral setae (Fig. 1E). Acrostichal setae absent, represented only by fine setulae; setulae uniserial presuturally, postsuturally uniserial anteriorly and becoming biserial posteriorly, with a few additional setulae between acrostichal and dorsocentral rows; the longest one about 0.4–0.55 length of longest intra-alar seta (Fig. 1E). Dorsocentral setae one or two presutural and three postsutural; when two presutural setae present, anterior one fine and less than half length of posterior one (Fig. 1E). Intra-alar seta absent presuturally, one postsutural, with a few accessory setulae. Pre-alar seta absent; one supra-alar seta. Two postpronotal setae (Fig. 1E). Two notopleural setae, without accessory setulae (Fig. 1D and E). Scutellum with one basal and one apical pair of setae, subequal in length, apical setae cruciate. Postalar declivity and suprasquamal ridge bare (Fig. 1B and E). Prosternum bare. Two proepisternal setae, directed upwards; two proepimeral setae, one directed upwards and one downwards, with surrounding setulae. Katepisternal setae 1:1:1. Posterior spiracle covered with dense light-yellow setulae, except dorsally (Fig. 1D).

Wing (Fig. 1A–D). Hyaline, slightly yellowish. Tegula and basicosta yellow (Fig. 1D). Vein R4+5 bare on both surfaces; vein M straight apically. Halter yellow (Fig. 1A). Calypters whitish-yellow; upper calypter short, distinctly shorter than wide; lower calypter tongue-shaped, about twice as long as upper calypter (Fig. 1D).

Legs (Fig. 1A). Yellow, concolorous with thorax. Fore femur with short anteroventral setal row on basal half, posterodorsal row on apical two-thirds, posterior row on basal one-thirds and a series of robust posteroventral setae throughout. Fore tibia with one apical dorsal seta. Mid-femur with short anteroventral and anterior setal rows on basal half, two strong posteroventral setae on basal half and one pre-apical posterodorsal seta. Mid-tibia with two posterodorsal setae, about half as long as first tarsomere. Hind femur with anteroventral row including two stronger setae on basal half (strongest at mid-length), shorter setae on apical half; anterodorsal row present, two posteroventral setae and one pre-apical anteroventral, one anterodorsal and one posterodorsal seta. Hind tibia with one small anteroventral seta on apical one-third, two anterodorsal and two posterodorsal setae on basal half and one pre-apical dorsal seta.

Abdomen (Fig. 1A and B). Conical in dorsal view, cylindrical in lateral view; concolorous with thorax, apical ground colour sometimes darkened. Median orange longitudinal vitta present on tergites 1+2–4. Paired oval black spots present on syntergite 1+2 to tergite 5, faint on syntergite 1+2, sometimes faint on tergite 3 and distinct on tergites 4–5. (Fig. 1B). Epandrium broad and subquadrate in posterior view, bearing dense long setae mainly along posterior margin; cerci small, elongate, lanceolate, with broadened basal portion; surstylus stout, apex inwardly curved and tapered and bearing apical tuft of setae in dorsal view (Fig. 1G–H). Sternite 5 relatively broad, shallowly emarginate medially, with elongate lobes densely setulose along inner margins (Fig. 1I–J). Pregonite short and lobe-like, with rounded apex; postgonite distinctly elongate and slender, weakly falcate, with acute apex curved ventrally; basiphallus well developed and subtriangular. Distiphallus elongate and strongly curved, with distinct digitiform basal process; apex hooked and recurved (Fig. 1F).

**Figure 2.**
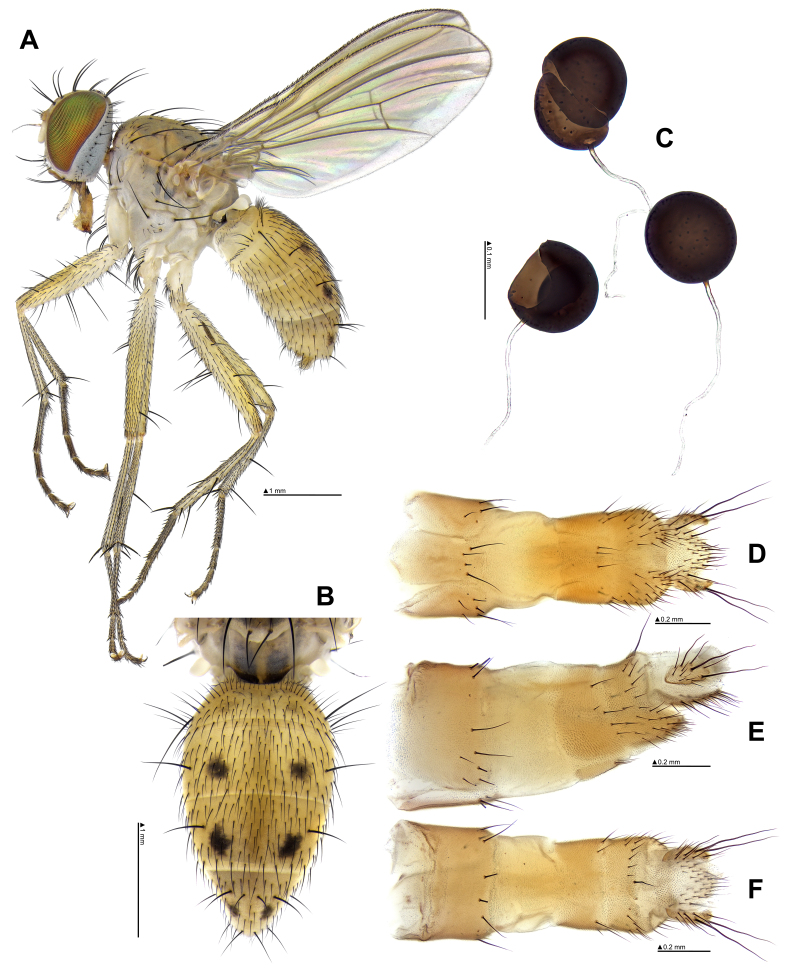
*Cephalispa acerca* (Xue, Wang & Ni, 1990), female. **A** habitus; **B** abdomen, dorsal view; **C** spermathecae; **D–F** female terminalia; ventral view (D), lateral view (E) and dorsal view (F).

**Figure 3.**
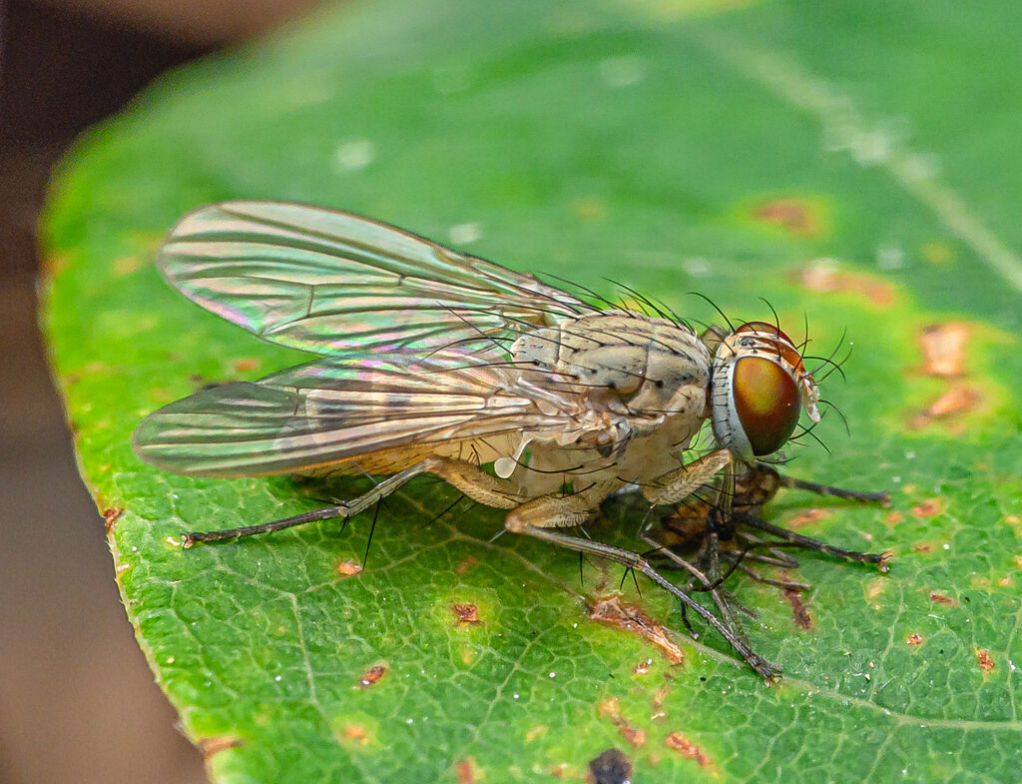
*Cephalispa acerca* (Xue, Wang & Ni, 1990), female preying on a smaller nematoceran fly (Mt. Muhak, Changwon-si, 1 June 2024).

**Female**. Body length 4.8–5.0 mm; wing length 4.2–4.3 mm (Fig. 2A and Fig. 3).

Similar to male in general appearance. Abdomen entirely yellow and more distinctly conical in lateral view (Fig. 2B). Spermathecae externally spherical, without modification; internally with numerous spinule-like projections (Fig. 2C). Cerci short, apically rounded and densely setulose. Abdominal tergites and sternites weakly sclerotised, with distinct marginal setae (Fig. 2D–F).

#### Diagnosis

Body yellow to beige; thoracic dorsum black, covered with yellowish-white pruinosity; scutum with three indistinct longitudinal brown vittae. Frons narrow, approximately one-fourth of head width at vertex (Fig. 1A and E); ocellar setae absent; arista shortly plumose (Fig. 1D). Acrostichal setae absent, represented only by fine setulae; dorsocentral setae one or two presutural and three postsutural (Fig. 1E); katepisternal setae 1+1+1 (Fig. 1D). Fore tibia lacking a submedian posterior or posteroventral seta; mid-tibia with two posterodorsal setae (Fig. 1A).

#### Distribution

Palaearctic Region: Korea (new record), China (Liaoning, Shaanxi, Shanxi).

#### Biology

The biology and ecology of *Cephalispa* are poorly known (Xue and Chao 1996). During the present study, predatory behaviour was repeatedly observed under natural conditions. Individuals were found resting on the surfaces of low-growing herbaceous leaves in forested habitats, from which they appeared to ambush potential prey. When small insects landed on adjacent or overlying leaves, the flies rapidly leapt upward to capture them.

Predation on small nematocerous flies (Diptera, Nematocera) was directly observed (Fig. 3), indicating that these insects constitute at least part of the prey spectrum. This observation suggests that *C. acerca* functions as an active predator within forest ecosystems.

The observed ambush strategy likely represents an adaptation to structurally complex understorey environments, where vertical movement between leaves may enhance prey detection and capture efficiency. Such behaviour is consistent with a sit-and-wait predatory mode combined with short-distance pursuit.

Predatory behaviour is a well-known ecological trait within Coenosiinae, a subfamily characterised by diverse hunting strategies and habitat use. Larvae of many coenosiine species develop in substrates such as moss, decaying vegetation or moist sand, reflecting broader ecological tolerance compared to other muscid groups (Gregor et al. 2002, Werner et al. 2014). In this context, the predatory behaviour observed in *C. acerca* is consistent with the ecological versatility of the subfamily.

To our knowledge, this is the first documented evidence of predatory behaviour, providing new insight into its ecological role. Further studies are required to clarify prey preferences, life history traits and the ecological significance of the genus within forest ecosystems.

#### Notes

Korean vernacular name : 연노랑범집파리(Yeon-no-rang-beom-jip-pa-ri) (신칭).

## Identification Keys

### Key to the males of East Asian *Cephalispa*.

**Table:** 

1	Mid-tibia with one posterodorsal seta	*Cephalispa mira* (Stein, 1900)
–	Mid-tibia with two posterodorsal setae	2
2	Lateral lobes of sternite 5 with dense, strong spines; frontal setae with three pairs of inclinate setae	*Cephalispa spina* Cui & Xue, 1995
–	Lateral lobes of sternite 5 without dense, strong spines; frontal setae with two or three pairs of inclinate setae	3
3	Fore tibia with a conspicuous submedian posterior seta	*Cephalispa biloba* Cui & Xue, 1995
–	Fore tibia lacking a submedian posterior seta	4
4	Ventral surfaces of femora on basal halves distinctly more setose than usual	*Cephalispa scutellata* (Malloch, 1935)
–	Ventral surfaces of femora with normal vestiture	5
5	Costa with ventral hairs; veins R4+5 and M1 apically curved posteriorly	*Cephalispa leptysmosa* Wei & Yang, 2007
–	Costa without ventral hairs; veins R4+5 and M1 straight	6
6	Sternite 5 with a pair of large, downward-curved processes above lateral lobes	7
–	Sternite 5 without distinct processes	8
7	Apex of sternite 5 processes expanded, nearly circular in lateral view	*Cephalispa prominentia* Cui & Xue, 1995
–	Apex of sternite 5 processes not expanded	*Cephalispa hamata* Cui & Xue, 1995
8	Acrostichal setae arranged in two distinct rows	9
–	Acrostichal setae absent or irregular, not forming distinct rows	12
9	Postpedicel short, about 1.6 times pedicel; abdomen without median vitta	*Cephalispa brevilamina* Cui & Xue, 1995
–	Postpedicel at least twice as long as pedicel; abdomen with median vitta	10
10	Abdomen long, cylindrical; tergite 7 without spots	*Cephalispa fujianensis* Cui & Xue, 1995
–	Abdomen short, oval; tergite 7 with lateral spots	11
11	Sternite 5 lobes triangular; antenna yellow	*Cephalispa cultrata* Cui & Xue, 1995
–	Sternite 5 lobes rectangular; antenna reddish-yellow	*Cephalispa breviabdominis* Cui & Xue, 1995
12	Postpedicel partly darkened	13
–	Postpedicel entirely pale	14
13	Head dark; thorax uniformly dark	*Cephalispa kudoi* Yoshizawa & Tachi, 2016
–	Head pale; thorax yellowish with reduced dark markings	*Cephalispa leigongshana* Wei & Yang, 2007
14	Thorax mostly yellow	15
–	Thorax mostly dark	17
15	Thorax without distinct dark patches; sternite 5 rounded	*Cephalispa acerca* (Xue, Wang & Ni, 1990)
–	Thorax with dark patches; sternite 5 elongate	16
16	Postpedicel about 2.7 times as long as wide; meron without spots	*Cephalispa apinesa* Wei & Yang, 2007
–	Postpedicel about 2.2 times as long as wide; meron with spots	*Cephalispa qiana* Wei & Yang, 2007
17	Sternite 5 lobes extremely narrow	*Cephalispa fani* Cui & Xue, 1995
–	Sternite 5 lobes broad	18
18	Sternite 5 rounded posteriorly	*Cephalispa xanthogaster* (Shinonaga, 2003)
–	Sternite 5 not rounded posteriorly	19
19	Sternite 5 curved dorsally posteriorly	*Cephalispa okinawaensis* Yoshizawa & Tachi, 2016
–	Sternite 5 nearly straight posteriorly	*Cephalispa triquetra* Yoshizawa & Tachi, 2016

(Modified from Cui et al. (1995), Yoshizawa and Tachi (2016)).

## Discussion

The genus *Cephalispa* currently comprises 32 described species worldwide, with the majority distributed in the Oriental and Afrotropical Regions, particularly Madagascar (Cui et al. 1995, Couri et al. 2006, Wei and Yang 2007, Yoshizawa and Tachi 2016). Species diversity is concentrated in southern China, particularly Guizhou and Yunnan and Southeast Asia including Malaysia and Indonesia, whereas records from the Palaearctic Region remain comparatively sparse (Cui et al. 1995, Wei and Yang 2007). The discovery of *C. acerca* in Korea represents the first record of *Cephalispa* from the country.

**Figure 4.**
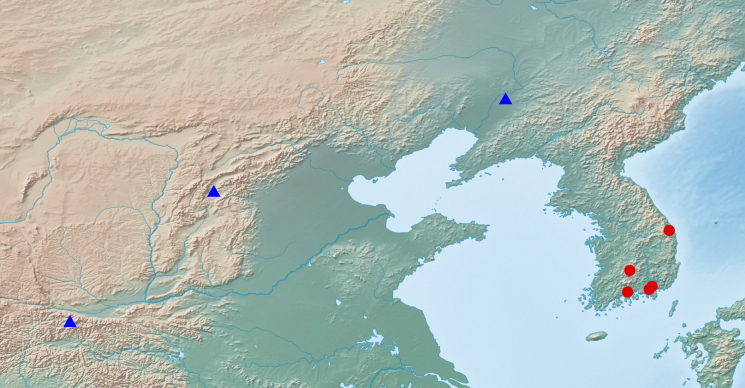
Distribution records of *Cephalispa acerca* (Xue, Wang & Ni, 1990). Red circles represent new records reported in the present study and blue triangles represent previously published records. Previous records are plotted approximately at the geographic centres of the reported regions because precise collecting localities were unavailable.

The distribution of *C. acerca* was previously known only from Liaoning, Shaanxi and Shanxi Provinces in China (Xue et al. 1989, Cui et al. 1995). The Korean records extend the known distribution of the species to the Korean Peninsula (Fig. 4). Together with records of other *Cephalispa* species from Japan, including *C. triquetra* and *C. xanthogaster* (Shinonaga 2003, Yoshizawa and Tachi 2016), this finding indicates that the genus is more widely distributed in temperate East Asia than previously recognised. While many *Cephalispa* species are restricted to tropical regions, including island systems such as Palau, Papua New Guinea and Madagascar (Snyder 1965, Couri et al. 2006), *C. acerca* appears to be associated with temperate forest habitats on the East Asian mainland. The geographic proximity of Liaoning to the Korean Peninsula suggests that the species may have a continuous distribution across north-eastern Asia, which has likely remained undocumented due to insufficient sampling.

This study also provides the first description of the female of *C. acerca*. As many species of *Cephalispa* have been described primarily, based on male specimens (Cui et al. 1995, Wei and Yang 2007), the inclusion of female characters, including external morphology and the structure of the abdominal terminalia, represents an important contribution to the taxonomy of the genus. These data will facilitate more reliable identification in future faunistic and ecological studies, particularly when male specimens are unavailable.

Despite its broad geographic distribution, molecular data for *Cephalispa* remain scarce. Searches of BOLD Systems and GenBank (accessed 13 July 2026) found no publicly available sequence linked to a species-level identification. BOLD contains 180 public specimen records representing 181 sequences from Malaysia, Indonesia and Thailand, all identified only to genus, whereas GenBank contains five loci (28S, 12S, 16S, COI and CYTB) from a single voucher identified as *Cephalispa* sp. SNK-2014 (RMBR:116183). Species-level molecular identification, therefore, cannot yet be reliably assessed. Broader sampling, voucher-based identification and validated reference sequences are needed for future molecular and integrative studies of the genus.

Field observations revealed predatory behaviour in *C. acerca*, representing the first ecological information reported for the genus. This behaviour was repeatedly observed under natural conditions, with individuals adopting an ambush strategy on low-growing herbaceous leaves and capturing small insects through rapid upward movements. Predation on nematocerous flies was directly observed, indicating that they form at least part of the prey spectrum. Although predatory behaviour is well documented in Coenosiinae (Gregor et al. 2002, Werner et al. 2014), the present observations provide the first evidence of such behaviour in *Cephalispa*.

In conclusion, the addition of *Cephalispa* to the Korean fauna, together with the first description of the female and the first ecological observations for the genus, expands current knowledge of the diversity, distribution, taxonomy and biology of *Cephalispa* in East Asia. Further studies integrating broader geographic sampling with morphological and molecular data will be important for clarifying species boundaries, evolutionary relationships and the biogeographic history of the genus.

## Supplementary Material

XML Treatment for Cephalispa

XML Treatment for Cephalispa acerca
